# Exploring the Concern about Food Allergies among Secondary School and University Students in Ontario, Canada: A Descriptive Analysis

**DOI:** 10.1155/2017/2051916

**Published:** 2017-05-16

**Authors:** Shannon E. Majowicz, James K. H. Jung, Sarah M. Courtney, Daniel W. Harrington

**Affiliations:** ^1^School of Public Health and Health Systems, University of Waterloo, 200 University Ave. West, Waterloo, ON, Canada N2L 3G1; ^2^Public Health Ontario, Suite 300, 480 University Avenue, Toronto, ON, Canada M5G 1V2

## Abstract

Our objective was to explore the perceived risk of food allergies among students in Ontario, Canada. We analyzed blinding questions (“I am concerned about food allergies”; “food allergies are currently a big threat to my health”) from three existing food safety surveys, given to high school and university undergraduate students (*n* = 3,451) circa February 2015, using descriptive analysis, and explored how concern related to demographics and self-reported cooking ability using linear regression. Overall, high school students were neutral in their concern, although Food and Nutrition students specifically were significantly less concerned (*p* = 0.002) than high school students overall. University undergraduates were moderately unconcerned about food allergies. Concern was highest in younger students, decreasing between 13 and 18 years of age and plateauing between 19 and 23 years. Among students aged 13–18 years, concern was higher among those who worked or volunteered in a daycare and who had previously taken a food preparation course. Among students aged 19–23 years, concern was higher among females and those with less advanced cooking abilities. Concern was significantly correlated with perceiving food allergies as a personal threat. This study offers a first exploration of perceived risk of food allergies among this demographic and can guide future, more rigorous assessments.

## 1. Introduction

Food allergies are a growing public health concern in many countries [1]. In Canada, the prevalence of food allergies is estimated to be 7.5 percent, as estimated by objective measures [2], yet almost 20 percent of individuals self-report having a food allergic person living in their home, and an additional 30 percent report being indirectly affected by food allergies, including serving, preparing, or buying foods for allergic individuals or for an allergen-controlled environment, such as a school with a peanut ban [1]. Previous research has identified particular groups who are more concerned than others, namely, females, older adults, recent immigrants to Canada, and those with lower levels of education [1]. Others have examined the management and experience of risks from the perspective of food allergic children [3, 4] and adolescents [5] and other vulnerable populations including low income families [6] and recent immigrants [7, 8].

Youth and young adults are a unique group in that they are generally growing in autonomy from their parents and increasingly making independent decisions about how to behave with respect to risks; in the realm of food, for example, youth and young adults often make risky food consumption and food handling choices [9–13]. Those with food allergies among this group are likely to engage in risk taking with respect to their allergies (e.g., not carrying an epinephrine autoinjector [14]). There is also an increased likelihood that youth and young adults are indirectly affected by food allergies as they take on responsibilities that may have previously been the purview of parents or caregivers, including buying, preparing, or serving foods to others that may have food allergies. These interactions may occur in their everyday lives (e.g., cooking at home for friends, eating out at restaurants), at school (e.g., in cafeterias), or in places of employment and volunteering (e.g., daycares and hospitals).

In the province of Ontario, Canada, the transition from elementary school to secondary school through to postsecondary school is important in the context of food allergies. Following the enactment of Sabrina's Law [15] in 2006, all publicly funded elementary and secondary schools in Ontario are mandated to train and educate staff about the risks of allergic reactions and are accountable for the management of pupils with severe (i.e., anaphylactic) allergies. However, there is evidence to suggest that while fatal allergic reactions do occur in schools [16], policies are more lenient in high schools in comparison with elementary schools [4] and that there is a relative paucity of support for food allergic individuals at the postsecondary level [17]. The result is that youth and young adults are interacting with foods in increasingly risky places with respect to the control of allergens and treatment of allergic reactions. Such changes in the policy environment may have important implications for how the risks of food allergies are understood by this group [18].

We argue that youth and young adults are important actors in the management of the risks of food allergies; however, little is known about the perception of the risks of food allergies in this group. Towards informing policy responses to food allergies, the goal of this study was to explore perceived risk of food allergies among the youth and young adult students at different academic stages. Specifically, we explored food allergy-related concern among high school and university students in Ontario, Canada, by conducting a secondary analysis of blinding questions from three surveys related to food safety more generally.

## 2. Materials and Methods

We analyzed data from three separate surveys (Table 1), conducted from late November 2014 to February 2015 in Ontario, Canada, that investigated the food safety knowledge and attitudes of students from four high schools (both among all students [19] and among Food and Nutrition students specifically [20]) and one university [21]. All had received ethics clearance through a University of Waterloo Research Ethics Committee. All three surveys had included blinding question(s) about food allergies, which were modified versions of questions previously developed to assess foodborne disease concern among college students [22], with the term “food poisoning” replaced with “food allergies.”

Our outcome of interest was students' “stated concern” about food allergies, measured via the question “I am concerned about food allergies” (5-point scale: 1-strongly disagree, 5-strongly agree) and analyzed as a continuous measure [23]. We also explored students' belief that “food allergies are currently a big threat to my health” (5-point scale: 1-strongly disagree, 5-strongly agree), which we considered a proxy for whether the respondent considered themselves food allergic. In addition to the allergy questions, the surveys also collected nine student-level covariates included in this analysis: age (in years); sex; whether or not they worked or volunteered in a hospital, restaurant/deli/other food service location, daycare or other location in which they interacted with children, or retirement home/nursing home/long-term care facility; whether or not they handled or prepared food in any of the aforementioned locations; whether or not they had ever taken a course where they were taught to prepare food or meals (e.g., high school food courses, food handler certification); and their self-described cooking ability (“I don't know how to cook”; “I can only cook food when the instructions are on the box (like Kraft Dinner®)”; “I can do basics from scratch (like boil an egg or make a grilled cheese sandwich) but nothing more complicated”; “I can prepare simple meals if I have a recipe to follow”; and “I can cook almost anything”). We included self-described cooking ability because we hypothesized that experience might mitigate concern, due to increased real or perceived control over one's meals and foods.

Data from the surveys were pooled and analyzed together. Student characteristics and food allergy perceptions were summarized descriptively. Differences in means were tested using *t*-tests with Bonferroni's adjustment for multiple comparisons. The nine covariate predictors of stated concern were assessed using linear multivariable regression modelling as follows. We first assessed the association between stated concern and each covariate individually, adjusting for survey source as a fixed effect. We then modelled the association between the nine potential predictors and stated concern, by first fitting the full model including all nine covariates and then removing any nonsignificant variables; survey source was retained in all models where possible, as a fixed effect regardless of significance. We then explored potential confounding by reintroducing any excluded variables and assessing whether the sign, significance, or magnitude of any regression coefficients changed meaningfully, retaining any variables for which this was the case. We then assessed all possible two-way interactions, for all significant variables in the final model. Data were analyzed using Stata/SE 14.1 for Mac (StataCorp, College Station, Texas).

## 3. Results

Student characteristics, their “stated concern” about food allergies, and their perception that “food allergies are currently a big threat to my health” are shown, by survey source and overall, in Table 2. Overall, high school students appeared neither concerned nor unconcerned about food allergies (mean: 3.1, standard deviation [SD]: 1.2), although those from Food and Nutrition courses specifically were significantly less concerned (mean: 2.7, SD: 1.2; *p* = 0.002) than high school students overall. University undergraduate students were moderately unconcerned (mean: 2.3, SD: 1.3) and significantly less concerned than Food and Nutrition students (*p* = 0.03).

**Figure 1:**
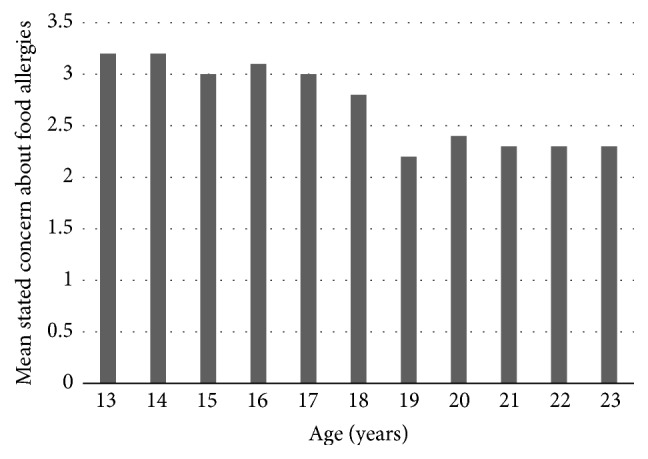
Mean agreement with the statement “I am concerned about food allergies” (“stated concern,” 5-point scale; 1-strongly disagree to 5-strongly agree) by age, among high school and university undergraduate participants (Ontario, Canada), circa February 2015 (*n* = 3,451).

The associations between individual predictors and stated concern (adjusting for survey source) are shown in Table 3; being female, working, or volunteering in a daycare or other location where they interact with children, handling food for the public, and having previously taken a food preparation course were all individually significantly associated with greater stated concern (adjusting for survey source). The relationship between stated concern and age varied depending on whether students were younger or older (Figure 1). Students aged 13 expressed the greatest, albeit still slight, stated concern about food allergies; between the ages of 13–18, stated concern decreased significantly by 0.059 points out of 5 for each year of age increase (adjusting for survey source; *p* = 0.001), whereas for students aged 19 and over, age and stated concern were not significantly associated (adjusting for survey source; *p* = 0.999).

Because stated concern varied with age, multivariable analyses of this outcome were stratified over two age groups: “13–18 years” (which included students from all three surveys) and “19–23 years” (which included university students only). Results of the final multivariable model for stated concern for students aged 13–18 are shown in Table 4. Among students aged 13–18, Food and Nutrition students and university students were significantly less concerned than high school students overall, and students who had taken a food preparation course, or who worked or volunteered in a location such as a daycare where they interacted with children, were significantly more concerned than students who did not, adjusting for the other factors in the model. Older students were less concerned about food allergies than younger students, regardless of whether they were in high school or university, with stated concern decreasing by 0.05 points out of 5 for each year of increase in age, adjusting for the other factors in the model.

Results of the final multivariable model for stated concern for students aged 19–23 are shown in Table 5. Among students aged 19–23, females were more concerned than males; those who reported that they “can do basics from scratch (like boil an egg or make a grilled cheese sandwich) but nothing more complicated” or who reported that they “can cook almost anything” were significantly less concerned than those who reported that they “don't know how to cook” (adjusting for the other factors in the model).

**Figure 2:**
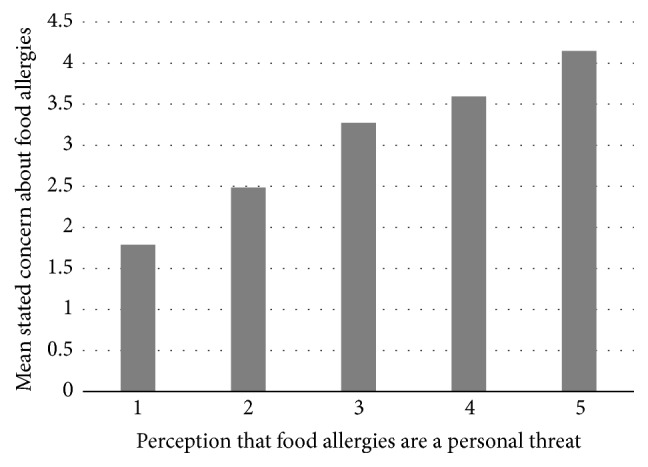
Mean agreement with the statement “I am concerned about food allergies” (“stated concern,” 5-point scale; 1-strongly disagree to 5-strongly agree), shown by whether students felt “food allergies are currently a big threat to my health” (5-point scale; 1-strongly disagree to 5-strongly agree) among high school and university undergraduate participants (Ontario, Canada), circa February 2015 (*n* = 515).

On average, Food and Nutrition students (mean: 2.2, SD: 1.3; Table 2) did not feel food allergies were a personal threat nor did university undergraduates (mean: 1.8, SD: 1.1), whose perception of food allergies as a personal threat was significantly lower than Food and Nutrition students (*p* = 0.001). Most students strongly disagreed (256/515; 49.7%) or disagreed (183/515; 35.5%), whereas 2.1% (11/515) were neutral, 7.2% (37/515) agreed, and 5.4% (28/515) strongly agreed with the statement “food allergies are currently a big threat to my health.” When examined individually (adjusting for survey source), sex was the only predictor significantly associated with the perception that food allergies are a personal threat, with this perception increased by 0.24 points out of 5 for females versus males (*p* = 0.047). In all of the multivariable models explored, sex remained the only significant predictor associated with the perception that food allergies were a personal threat, and this relationship did not change when potential confounders and interactions were included (i.e., the coefficient estimate and *p* value for the variable “sex” did not change meaningfully; results are not shown). Responses to the personal threat statement were significantly correlated with students' stated concern, among both Food and Nutrition (*r* = 0.48, *p* < 0.001) and university students (*r* = 0.55, *p* < 0.001), and mean stated concern increased linearly with perceiving allergies as a personal threat (Figure 2).

## 4. Discussion

This study explored the perceived risk of food allergies among high school and university undergraduate students in Ontario, Canada, using two blinding questions that had been previously collected via three surveys on food safety. Overall, students ranged from neither concerned nor unconcerned (high school students overall) to moderately unconcerned (university students). In addition to whether students were in high school or university, we found that older students were significantly less concerned about food allergies than younger students and that the association between other student characteristics and concern differed by age as follows.

Here, students' stated concern about food allergies changed with age, with students aged 13 years old reporting the highest, yet still slight, concern about food allergies. Concern then decreased to being slightly unconcerned by the age of 18 and plateaued across ages 19–23. Without overstating the significance of this finding, it may be possible that these results reflect changes in feelings of personal vulnerability across adolescence. A previous study found that older adults have higher perceived concerns of food allergies than younger adults [1], but given the paucity of literature, the relationship between age and food allergy is currently not well understood and should be elucidated via future research.

We found that, among students aged 13 to 18 years, concern about food allergies was significantly greater among those who worked or volunteered in a daycare or other location involving children versus those who did not. This elevated concern may be due to increased awareness of, and exposure to, a subpopulation with a disproportionately high risk of food allergies (i.e., children [24, 25]) within settings in which food allergy policies are often clearly apparent and enforced (e.g., licensed childcare facilities). Students aged 13 to 18 also reported significantly greater concern about food allergies if they had taken a previous course in which they were taught to handle or prepare food; unfortunately, due to our cross-sectional study design we could not determine whether students with food allergy concerns go on to take food preparation courses, or whether those who take such courses become aware of food allergy issues thereby increasing their concern. In contrast to those aged 13 to 18 years, among students aged 19 to 23 years, better self-reported cooking abilities were associated with lower concern about food allergies. Although this study did not collect detailed information on the range and extent of students' food-related experience and skills, these results suggest that increased experience, both with cooking and with feeding oneself (which may or may not involve cooking), may be mitigating factors in the perceived risk of food allergies. The role such factors play in the perceived risk of food allergies, including the extent to which experience preparing foods provides a coping resource to youth and young adults, may be an important direction for future research. In addition, the role of the social amplification of risk [26], including how forces such as media coverage and socioeconomic and political factors influence risk perception among youth and young adults, bears investigating.

Here, among students aged 19–24, but not also among those aged 13–18, female students reported greater concern about food allergies than did male students. This is consistent with other studies that have identified a higher perceived risk of food allergy among females [1]. Reasons for gender differences in risk perception more generally are unclear and are thought to arise from a combination of biological and social differences [27] as well as from differences in self-efficacy and the general belief in an individual's own ability to respond to and control environmental demands [28]. Our analysis allowed us to explore the association between gender and concern, adjusting for several potentially important social factors, including self-reported cooking ability and whether the student had previously taken a food preparation or food handling course. We observed significantly greater concern among females than males even after accounting for the effects of these experiential factors, suggesting that gender differences in perceived food allergy risk may not relate strongly to gender differences in cooking ability or training. However, it is important to note that our sample size and limited measurement of cooking and experience factors may have been insufficient to fully elucidate such relationships and that future studies may wish to further explore relationships between gender, food allergy risk perception, and cooking abilities and experiences.

## 5. Limitations

This study is subject to several limitations. The most significant is that we did not know participants' allergy status and thus could not assess how stated concern related to, or varied by, actual food allergies. Rather, our measure of food allergy concern likely represents a composite of personal food allergy status (including both diagnosed and perceived allergies and their real or perceived severity), as well as the diagnosed and perceived food allergy status of those in the student's network of friends, family members, classmates, and others. Future studies should assess how food allergy concern relates to personal food allergy status and familiarity with food preparation practices, to help further elucidate these relationships. A second main limitation is that our data were cross-sectional; we could not discern the temporal sequence of the various factors we assessed and could not determine whether differences observed by age are due to age, period, or cohort effects. Future studies that follow a cohort of individuals over time are needed to more fully uncover how food allergy concern relates to physical age and birth cohort and life stage effects. Despite these limitations, this study is the first to focus on the perceived concerns of food allergies among high school and university undergraduate students in Canada and offers insight into the unique experiences of this population as they navigate increasingly risky environments with respect to food allergies.

## 6. Conclusions

This study found that, overall, high school and university undergraduate students in Ontario, Canada, are generally unconcerned about food allergies but that certain types of students, such as younger high school students and those who work or volunteer with children, as well as university students with less well-developed cooking abilities, are more concerned than others. Students who report that food allergies are a personal threat express the most concern. This first exploration can be used to guide future, more rigorous assessments of the perceived risk of food allergies in various related populations.

## Figures and Tables

**Table 1:** Descriptions of the three surveys whose data were used in this study.

	High school		University Student Survey [21]
	Whole High School Survey [19]	Food & Nutrition Class Survey [20]	
Study period	November 2014-December 2014	February 2015	February 2015
Study population	The whole student body of four Ontario high schools	Students in eight Food and Nutrition classes in the same four Ontario high schools	University of Waterloo undergraduate students
Survey method	Paper	Paper	Electronic
Sample size	2,860	106	485
Participation rate	79.1%	84.1%	9.7%

**Table 2:** Characteristics of the high school and university undergraduate participants (Ontario, Canada, circa February 2015) included in this study by survey source and pooled together.

	High school		University Student Survey [21] (*n* = 485)	Pooled data (*n* = 3,451)
	Whole High School Survey [19] (*n* = 2,860)	Food & Nutrition Survey [20] (*n* = 106)		
Mean age in years (SD)	15.5 (1.2)	15.7 (1.2)	20.5 (1.6)	16.2 (2.1)
% female	52.7	67.0	65.0	54.8
% of students who work or volunteer in				
Restaurant/food service location	16.8	21.7	10.1	16.0
Daycare	2.6	1.9	6.2	3.1
Hospital	11.7	16.0	8.5	11.4
Retirement home/long-term care facility	4.9	4.7	1.9	4.5
% of students who handle food for the public in any of the above settings	18.0	26.4	10.6	17.2
% of students who had previously taken a food preparation course	32.8	32.1	41.1	35.7
% of students by self-described cooking ability				
“Don't know how”	5.5	3.9	0.6	4.9
“Can only cook when the instructions are on the box”	10.0	10.7	2.3	9.2
“Can do the basics from scratch (like boil an egg)…”	17.2	12.6	6.7	16.1
“Can prepare simple meals if they have a recipe to follow”	42.8	50.5	50.6	45.4
“Can cook almost anything”	20.9	22.3	39.8	24.3
Mean food allergy perceptions (SD)^a^				
“I am concerned about food allergies” (stated concern)	3.1 (1.2)	2.7 (1.2)	2.3 (1.3)	3.0 (1.2)
“Food allergies are currently a big threat to my health”	—^b^	2.2 (1.3)	1.8 (1.1)	1.8 (1.1)

^a^1-strongly disagree to 5-strongly agree. ^b^This question was not asked in this survey.

**Table 3:** Individual associations between student characteristics and stated concern about food allergies (1-strongly disagree to 5-strongly agree) among high school and university undergraduate participants (Ontario, Canada), circa February 2015 (each adjusting for survey source; *n* = 3,451); 95% confidence intervals (CIs) that do not contain the value “1” are shown in bold.

	Coefficient	95% CI
Age (in years)	−0.03	−0.06, 0.01
Gender (referent: male)	**0.11**	**0.03, 0.20**
Works or volunteers in		
Restaurant/other food service location	0.03	−0.08, 0.14
Daycare	**0.18**	**0.05, 0.31**
Hospital	0.01	−0.23, 0.24
Retirement home/long-term care facility	−0.01	−0.20, 0.19
Handles food for the public in any of the above settings	**0.12**	**0.01, 0.23**
Had previously taken a food preparation course	**0.17**	**0.08, 0.26**
Self-described cooking ability (referent: “Don't know how to cook”)		
“Can only cook when the instructions are on the box”	−0.13	−0.37, 0.10
“Can do the basics from scratch (like boil an egg)…”	−0.18	−0.40, 0.04
“Can prepare simple meals if they have a recipe to follow”	−0.13	−0.33, 0.07
“Can cook almost anything”	−0.71	−0.28, 0.14

**Table 4:** Factors associated with stated concern about food allergies (1-strongly disagree to 5-strongly agree) among students aged 13–18 years old, circa February 2015 (final multivariable regression model; *n* = 2,844); 95% confidence intervals (CIs) that do not contain the value “1” are shown in bold.

	Coefficient	95% CI
Intercept	**3.84**	**3.27, 4.41**
Survey source (referent: Whole High School Survey)		
Food & Nutrition Survey	**−0.43**	**−0.66, −0.19**
University Student Survey	**−0.62**	**−0.93, −0.31**
Age (in years)	**−0.05**	**−0.09, −0.02**
Works or volunteers in a daycare	**0.18**	**0.05, 0.32**
Had previously taken a food preparation course	**0.22**	**0.13, 0.32**

**Table 5:** Factors associated with stated concern about food allergies (1-strongly disagree to 5-strongly agree) among students aged 19–23 years old, circa February 2015 (final multivariable regression model; *n* = 420); 95% confidence intervals (CIs) that do not contain the value “1” are shown in bold.

	Coefficient	95% CI
Intercept	**3.49**	**2.06, 4.92**
Gender (referent: male)	**0.28**	**0.01, 0.55**
Self-described cooking ability (referent: “Don't know how to cook”)		
“Can only cook when the instructions are on the box”	−0.92	−2.51, 0.66
“Can do the basics from scratch (like boil an egg)…”	**−1.58**	**−3.11, −0.05**
“Can prepare simple meals if they have a recipe to follow”	−1.25	−2.67, 0.18
“Can cook almost anything”	**−1.46**	**−2.89, −0.03**
Works or volunteers in a restaurant or other food service location	−0.72	−1.71, 0.27
Handles food for the public in any of the above settings	0.56	−0.39, 1.52
Had previously taken a food preparation course	−0.03	−0.29, 0.23

## References

[1] Harrington D. W., Elliott S. J., Clarke A. E., Ben-Shoshan M., Godefroy S. (2012). Exploring the determinants of the perceived risk of food allergies in Canada. *Human and Ecological Risk Assessment*.

[2] Soller L., Ben-Shoshan M., Harrington D. W. (2015). Adjusting for nonresponse bias corrects overestimates of food allergy prevalence. *Journal of Allergy and Clinical Immunology: In Practice*.

[3] Fenton N. E., Elliott S. J., Cicutto L., Clarke A. E., Harada L., McPhee E. (2011). Illustrating risk: anaphylaxis through the eyes of the food-allergic child. *Risk Analysis*.

[4] Dean J., Fenton N. E., Shannon S., Elliott S. J., Clarke A. (2016). Disclosing food allergy status in schools: health-related stigma among school children in Ontario. *Health and Social Care in the Community*.

[5] Protudjer J. L. P., Jansson S. A., Middelveld R. (2016). Impaired health-related quality of life in adolescents with allergy to staple foods. *Clinical and Translational Allergy*.

[6] Minaker L. M., Elliott S. J., Clarke A. (2014). Exploring low-income families’ financial barriers to food allergy management and treatment. *Journal of Allergy*.

[7] Harrington D. W., Dean J., Wilson K., Qamar Z. (2015). "We don't have such a thing, that you may be allergic": newcomers' understandings of food allergies in Canada. *Chronic Illness*.

[8] Lu S. K., Elliot S. J., Clarke A. E. (2014). Exploring perceptions and experiences among new Canadians from Asia. *Journal of Allergy*.

[9] Abbot J. M., Policastro P., Bruhn C., Schaffner D. W., Byrd-Bredbenner C. (2012). Development and evaluation of a university campus-based food safety media campaign for young adults. *Journal of Food Protection*.

[10] Byrd-Bredbenner C., Maurer J., Wheatley V., Cottone E., Clancy M. (2007). Observed food safety behaviours of young adults. *British Food Journal*.

[11] Morrone M., Rathbun A. (2003). Health education and food safety behavior in the university setting. *Journal of Environmental Health*.

[12] Nesbitt A., Majowicz S., Finley R. (2009). High-risk food consumption and food safety practices in a Canadian community. *Journal of Food Protection*.

[13] Stein S. E., Dirks B. P., Quinlan J. J. (2010). Assessing and addressing safe food handling knowledge, attitudes, and behaviors of college undergraduates. *Journal of Food Science Education*.

[14] Sampson M. A., Muñoz-Furlong A., Sicherer S. H. (2006). Risk-taking and coping strategies of adolescents and young adults with food allergy. *Journal of Allergy and Clinical Immunology*.

[15] Bill 3 Sabrina's law.

[16] Bock S. A., Muñoz-Furlong A., Sampson H. A. (2007). Further fatalities caused by anaphylactic reactions to food, 2001–2006. *Journal of Allergy and Clinical Immunology*.

[17] Olarnyk A. S., Elliott S. J. (2016). "You're totally on your own": experiences of food allergy on a Canadian university campus. *Universal Journal of Public Health*.

[18] Harrington D. W., Elliott S. J. (2015). Understanding emerging environmental health risks: A framework for responding to the unknown. *Canadian Geographer*.

[19] Majowicz S. E., Diplock K. J., Leatherdale S. T. (2015). Food safety knowledge, attitudes and self-reported practices among Ontario high school students. *Canadian Journal of Public Health*.

[20] Majowicz S. E., Hammond D., Dubin J. A. (2017). A longitudinal evaluation of food safety knowledge and attitudes among Ontario high school students following a food handler training program. *Food Control*.

[21] Courtney S. M., Majowicz S. E., Dubin J. A. (2016). Food safety knowledge of undergraduate students at a Canadian university, results of an online survey. *BMC Public Health*.

[22] Byrd-Bredbenner C., Maurer J., Wheatley V., Schaffner D., Bruhn C., Blalock L. (2007). Food safety self-reported behaviors and cognitions of young adults: results of a national study. *Journal of Food Protection*.

[23] Norman G. (2010). Likert scales, levels of measurement and the 'laws' of statistics. *Advances in Health Science Education: Theory and Practice*.

[24] Sicherer S. H., Muñoz-Furlong A., Sampson H. A. (2003). Prevalence of peanut and tree nut allergy in the United States determined by means of a random digit dial telephone survey: a 5-year follow-up study. *Journal of Allergy and Clinical Immunology*.

[25] Ben-Shoshan M., Harrington D. W., Soller L. (2012). Demographic predictors of peanut, tree nut, fish, shellfish, and sesame allergy in Canada. *Journal of Allergy*.

[26] Renn O., Burns W. J., Kasperson J. X., Kasperson R. E., Slovic P. (1992). The social amplification of risk: theoretical foundations and empirical applications. *Journal of Social Issues*.

[27] Finucane M. L., Slovic P., Mertz C. K., Flynn J., Satterfield T. A. (2010). Gender, race, and perceived risk: the 'white male' effect. *Health, Risk and Society*.

[28] DunnGalvin A., Hourihane J. O., Frewer L., Knibb R. C., Oude Elberink J. N. G., Klinge I. (2006). Incorporating a gender dimension in food allergy research: a review. *Allergy*.

